# The distribution of pathogens and their antimicrobial susceptibility patterns among canine surgical wound infections in Sweden in relation to different risk factors

**DOI:** 10.1186/s13028-015-0102-6

**Published:** 2015-02-28

**Authors:** Ulrika Windahl, Björn Bengtsson, Ann-Kristin Nyman, Bodil Ström Holst

**Affiliations:** Department of Animal Health and Antimicrobial Strategies, National Veterinary Institute, SVA, Uppsala, SE-75189 Sweden; Department of Clinical Sciences, Swedish University of Agricultural Sciences, Uppsala, SE-75007 Sweden

**Keywords:** Surgical site infection, Bacterial infection, Canine, Dog, Antimicrobial resistance

## Abstract

**Background:**

Surgical site infection (SSI) is a common nosocomial infection in dogs and a growing concern in veterinary hospitals as an increase in multidrug-resistant pathogens is reported. Despite the need for rational and prudent antimicrobial use, few peer-reviewed and published veterinary studies have investigated the pathogenic growth including susceptibility patterns of the isolated pathogens in canine SSIs.

The first objective of the present study was to estimate the distribution of bacterial pathogens in dogs with SSI and to investigate whether this was influenced by type of surgical procedure (clean, clean-contaminated, contaminated or dirty), duration of hospitalization, wound classification and depth of the infection, or antimicrobial treatment. The second objective was to assess susceptibility patterns to clinically relevant antimicrobials.

During three years, four animal referral hospitals and three small animal clinics submitted bacterial swabs from canine SSIs for culture and susceptibility, together with a questionnaire completed by the attending clinician.

**Results:**

Approximately two thirds of the in total 194 isolates were staphylococci. *Staphylococcus pseudintermedius* was the most prevalent finding (46%) followed by beta haemolytic *Streptococcus* spp. (24%). No associations between distribution of the isolated pathogens and classification of the surgical procedure, duration of hospitalization or depth of the SSI were shown, with the exception of *Escherichia coli* isolates being significantly more often found in deep wound infections than in superficial skin infections.

Overall the possibilities of finding first generations antimicrobials to treat the SSIs included in the study were favorable, as the isolated pathogens were mostly without acquired antimicrobial resistance and multidrug resistance was uncommon. There were only three cases of methicillin-resistant *S. pseudintermedius*-infections (one percent of all isolates), one case of extended-spectrum beta-lactamase producing *E. coli*-infection, and no methicillin-resistant *Staphylococcus aureus* infections.

**Conclusions:**

None of the investigated factors were shown to influence the distribution of bacterial pathogens. The majority of SSIs were caused by staphylococci, and *S. pseudintermedius* was the most prevalent pathogen. Based on the study results, use of first-line antimicrobials prior to receiving culture and susceptibility results is a rational empirical antimicrobial therapy for the studied dog population.

## Background

Surgical site infections (SSIs) has been described as a complication of 0.8% to 18.1% of small animal surgical procedures [1]. Postoperative infections can cause increased patient morbidity, affect the success of initial surgical intervention, delay healing, and in veterinary medicine also cause additional costs for the animal owners. SSIs are a growing concern in veterinary hospitals as an increase in multidrug-resistant (MDR) canine pathogens is reported, including the emergence of multidrug resistant clones of methicillin-resistant *Staphylococcus pseudintermedius* in dogs, as well as with canine methicillin-resistant *Staphylococcus aureus* infections and Enterobacteriaceae resistant to extended spectrum cephalosporins (ESC) [1-6].

Surgical site infections cannot be completely eliminated, and preventive strategies represent the most economical and effective means of reducing their impact. Proper preventative measures include adherence to aseptic principles and good surgical techniques as well as proper preparing and caring of the dog and the surgical area prior to, during, and after surgery [1]. Development and implementation of proper infection control programs to prevent spread of antimicrobial resistant bacteria through clinics and hospitals, as well as surveillance protocols including patterns of antimicrobial resistance have in human medicine been shown to be key components in reducing infection rates [1,3,7]. Bacterial culture and antimicrobial susceptibility testing of clinical SSIs in veterinary practice is important for diagnosis of bacterial growth and to guide treatment towards rational and prudent antimicrobial use. Culture-based therapy also helps to facilitate surveillance efforts [1,8]. However, empirical treatment is often necessary, for example before culture and susceptibility results are available or when perioperative prophylactic antimicrobial therapy is needed.

The focus in veterinary studies on SSIs in dog has tended to be directed towards investigating possible risk factors for developing an infection, and few recent veterinary studies have investigated the pathogenic growth, including susceptibility patterns of the isolated pathogens in canine SSIs [2,8-18].

The first objective of the present study was to estimate the distribution of bacterial pathogens in dogs with SSI and to investigate whether this was influenced by type of surgical procedure (clean, clean-contaminated, contaminated or dirty as defined by the National Nosocomial Infections Surveillance classification scheme [7]), duration of hospitalization, wound classification and depth of the infection, or antimicrobial treatment.

The second objective was to assess susceptibility patterns to clinically relevant antimicrobials. Enterobacteriacae resistant to extended spectrum cephalosporins, methicillin-resistant *S. pseudintermedius* (MRSP), methicillin-resistant *S. aureus* (MRSP) and *Staphylococcus schleiferi* and multidrug resistant phenotypes were of particular interest.

The results of the study were intended to aid in assessing influence of the investigated factors on bacterial growth from SSIs in dogs in clinical practice and to provide a basis for rational empirical antimicrobial therapy, as well as to provide a basis for future monitoring of trends in antimicrobial resistance.

## Methods

### Design of the study

Four animal referral hospitals and three small animal clinics were enrolled in the study. They were asked to sample all detected SSIs in dogs at time of diagnosis during three years (April 1, 2008 - April 29, 2010) and immediately send the bacterial swabs by post to the National Veterinary Institute (SVA) for culture and susceptibility testing together with a questionnaire completed by the attending clinician.

The predetermined inclusion criteria were positive bacterial growth from a wound infection clinically diagnosed within a month after surgery and immediately cultured and submitted together with a completed questionnaire. For classification of the clinicians’ descriptions of the SSIs, the authors used the criteria developed by The Centers for Disease Control and Prevention National Nosocomial Infections Surveillance [1,7].

Data collected included surgical procedure, the date of surgery and of bacterial sampling, name of the referring clinic or hospital, whether the surgical site was visibly contaminated prior to surgery or not, if contamination during surgery was suspected, if a clinically evident infection was present prior to surgery or not, duration of hospitalization and of antimicrobial treatment, including substances used and dates of treatment. The veterinarians also subjectively described the SSI including how the diagnosis was made and location, including depth, of the infection.

The four referral hospitals were labelled submission category A, B, C and D. The three animal clinics were labelled submission category E. Two categories of hospitalization time were defined. Isolates from dogs with SSI that spent one day or less in the hospital or clinic were labeled hospitalization group one. The remaining isolates were labeled hospitalization group two. Each dog with SSI included in the study was defined as one case.

Based on the questionnaires, the surgical procedures were categorized as clean, clean-contaminated, contaminated or dirty according to the classification scheme developed by The Centers for Disease Control and Prevention National Nosocomial Infections Surveillance [7]. Surgical site infections described as superficial and only involving skin were labeled superficial skin infections, and all infections described as either deep or deep subcutaneous were labeled deeper, as were infections including soft or skeletal tissue and abscesses. Two categories of antimicrobial treatment were defined: treatment or no treatment. Within the treatment group, four categories were defined depending on whether the antimicrobial treatment was ongoing at time of sampling or not, and whether the isolate was susceptible or not to the antimicrobial used.

### Bacteriological examination

The obtained samples were inoculated and spread on horse-blood agar (SVA, Uppsala, Sweden) and on bromocresol purple lactose agar (SVA, Uppsala, Sweden) on the day of arrival. All inoculated agar plates were incubated at 37°C for 24 to 48 h until presence of adequate growth. Identification of pathogens was based on colony type and morphology, gram staining characteristics and standard biochemical tests [19-21]*.*

### Antimicrobial susceptibility testing

Isolated pathogenic bacteria were tested for susceptibility to antimicrobials relevant for the respective bacterial species by determination of minimum inhibitory concentration (MIC) by broth microdilution method using VetMIC panels (SVA, Uppsala, Sweden). The classification of susceptibility were performed using clinical breakpoints (BP) according to the standards of the Clinical and Laboratory Standards Institute (CLSI) [22] (Table 1). In addition, staphylococci were tested for beta-lactamase production by the “clover-leaf” method as described by Bryan and Godfrey [23]. *Escherichia coli* ATCC 25922, *Enterococcus faecalis* ATCC 29212, *S. aureus* ATCC 29213 and *Pseudomonas aeruginosa* ATCC 27853 were used for quality control.

**Table 1:** **Antimicrobial susceptibility of isolated pathogens in the canine surgical site infections**

		**Antimicrobials tested** ^**a**^										
		**AMP**	**PEN**	**CEP**	**OXA**	**CTX**	**ERY**	**CLI**	**ENR**	**GEN**	**TET**	**T-S**
***Staphylococcus pseudintermedius n = 90***	**BP-S**	ß-lact.^b^	ß-lact.^b^	≤2	≤0.25	NR^f^	≤0.5	≤0.5	≤0.5	≤2	≤4	≤0.5/9.5
	**% S**	20.0	20.0	95.6	NR^d^	NR^f^	76.7	NR^d^	94.4	93.3	73.3	75.6
	**MIC**_**50**_	NA^c^	NA^c^	≤2	≤0.5	NR^f^	≤0.5	≤1	≤0.12	≤2	≤1	≤0.5/9.5
	**MIC**_**90**_	NA^c^	NA^c^	2	≤0.5	NR^f^	>4	>4	0.25	≤2	>8	1/18
***Staphylococcus aureus n = 15***	**BP-S**	ß-lact.^b^	ß-lact.^b^	≤2	≤2	NR^f^	≤0.5	≤0.5	≤0.5	≤2	≤4	≤0.5/9.5
	**% S**	20.0	20.0	100	100	NR^f^	100	NR^d^	100	100	93.3	93.3
	**MIC**_**50**_	NA^c^	NA^c^	≤2	≤0.5	NR^f^	≤0.5	≤1	≤0.12	≤2	≤1	≤0.5/9.5
	**MIC**_**90**_	NA^c^	NA^c^	≤2	≤0.5	NR^f^	≤0.5	≤1	0.25	≤2	≤1	≤0.5/9.5
***Staphylococcus schleiferi coagulans n = 7***	**BP-S**	ß-lact.^b^	ß-lact.^b^	≤2	≤25	NR^f^	≤0.5	≤0.5	≤0.5	≤2	≤4	≤0.5/9.5
	**% S**	14.3	14.3	100	NR^d^	NR^f^	71.4	85.7	100	100	85.7	100
	**MIC**_**50**_	NA^c^	NA^c^	≤2	≤0.5	NR^f^	≤0.5	≤1	0.25	≤2	≤1	≤0.5/9.5
	**MIC**_**90**_	NR^e^	NR^e^	NR^e^	NR^e^	NR^f^	NR^e^	NR^e^	NR^e^	NR^e^	NR^e^	NR^e^
**beta haemolytic** ***Streptococcus*** **spp. n = 47**	**BP-S**	≤0.25	≤0.12	≤2	NR^f^	NR^f^	≤0.25	≤0.5	≤0.5	≤2	≤2	≤0.5/9.5
	**% S**	NR^d^	100	100	NR^f^	NR^f^	NR^d^	NR^d^	17.0	0.0	66.0	100
	**MIC**_**50**_	≤1	≤0.12	≤2	NR^f^	NR^f^	≤0.5	≤1	1	8	2	≤0.5/9.5
	**MIC**_**90**_	≤1	≤0.12	≤2	NR^f^	NR^f^	≤0.5	≤1	2	8	>8	≤0.5/9.5
***Escherichia coli n = 20***	**BP-S**	≤0.25	-^f^	≤2	NR^f^	≤1	NR^f^	NR^f^	≤0.5	≤2	≤4	≤0.5/9.5
	**% S**	NR^d^	-^f^	5.0	NR^f^	95.0	NR^f^	NR^f^	100	100	80.0	90.0
	**MIC**_**50**_	4	-^f^	>4	NR^f^	≤0.25	NR^f^	NR^f^	≤0.12	≤2	8	≤0.5/9.5
	**MIC**_**90**_	>16	-^f^	>4	NR^f^	≤0.25	NR^f^	NR^f^	≤0.12	≤2	16	≤0.5/9.5
***Pasteurella multocida n = 6***	**BP-S**	≤0.25	≤0.25	≤2	NR^f^	NR^f^	NR^f^	NR^f^	≤0.5	≤2	≤2	≤0.5/9.5
	**% S**	NR^f^	100	100	NR^f^	NR^f^	NR^f^	NR ^f^	100	100	100	100
	**MIC**_**50**_	≤1	≤0.12	≤2	NR^f^	NR^f^	2	≤1	≤0.12	≤2	≤1	≤0.5/9.5
	**MIC**_**90**_	NR^e^	NR^e^	NR^e^	NR^e^	NR^f^	NR^e^	NR^e^	NR^e^	NR^e^	NR^e^	NR^e^

Antimicrobial susceptibility presented as the percentage of susceptible isolates (%S) and as MIC_50_ and MIC_90_ values (mg/L), i.e. the MIC required to inhibit 50% and 90% of the isolates respectively. Breakpoints used to define isolates as susceptible (BP-S) are according to CLSI [22].

^a^Ampicillin (AMP), penicillin (PEN), cephalothin (CEP), oxacillin (OXA), cefotaxime (CTX), erythromycin (ERY), clindamycin (CLI), enrofloxacin (ENR ), gentamicin (GEN), tetracycline (TET), trimethoprim- sulfamethoxazole (T-S).

^b^Resistant to penicillin and ampicillin through beta-lactamase production (ß-lact).

^c^Not applicable (NA), as susceptibility is based on ß-lactamase production.

^d^Not relevant (NR) as breakpoints used to define isolates as susceptible (BP-S) [22] is below the range of concentrations tested.

^e^Not relevant (NR) as the small number of isolates precludes calculation of MIC_90_.

^f^Not relevant (NR) as no BP-S is available.

Isolates were classified as susceptible to an antimicrobial according to MIC BPs for bacteria from animals issued by CLSI [22]. Isolates of staphylococci spp. producing beta-lactamase were classified as resistant to ampicillin and penicillin. An isolate was classified as MDR if it was resistant to three or more antimicrobial classes to which the bacterial species is normally susceptible. For classification of MDR in staphylococci and streptococci, ampicillin, cephalothin and penicillin were considered as one antimicrobial class.

### Genotyping

Staphylococci were tested for susceptibility to oxacillin as indicator for methicillin resistance and isolates with elevated MICs were examined for presence of the *mec*A gene by PCR according to Strommenger *et al.* [24]. Moreover, *E. coli* and *Proteus mirabilis* were tested for susceptibility to cefotaxime as indicator for resistance to ESC. Isolates with cefotaxime MIC > 0.25 mg/L were examined for presence of genes coding for production of extended spectrum beta-lactamases (ESBL) (gene groups TEM, SHV, CTX-M-1, −3 and −9) or plasmid mediated AmpC beta-lactamases (gene groups MOX, CIT, DHA, ACC,EBC and FOX) by PCR [25-27]. Specific gene variants were determined by sequencing using in house primers and Big-Dye™ v1.1 [28].

### Statistical analyses

Descriptive statistics were performed to describe the distributions of the isolated pathogens within each of the categories of the explanatory factors as listed in Table 2. Univariable associations were investigated between findings of the isolated pathogens and the explanatory variables using Fisher’s exact-test, the χ^2^-test and univariable logistic regression analysis. Moreover, for every pathogen a multivariable logistic regression analysis was performed using a manual stepwise backward variable selection procedure where the initial models included all explanatory variables as main effects. Before inclusion in the multivariable models the collinearity between the explanatory variables was assessed pair-wise by Spearman rank correlations. If there was collinearity (r ≥ 0.70) the variable with lowest *P*-value in the univariable analysis was selected for further modeling. All plausible two-way interactions between the significant main effects were tested. Variables with a significant association (*P* < 0.05) with the dependent variable were kept in the model. The model fit was evaluated with the Hosmer-Lemeshow goodness-of-fit test and by visual examination of diagnostic plots as outlined by Hosmer and Lemeshow [29]. For all analyses, the level of statistical significance was set at *P* ≤ 0.05.

**Table 2:** **Isolated pathogens in the canine surgical site infections**

		***Staphylococcus pseudintermedius*** **(n)**	**beta haemolytic** ***Streptococcu*** **s spp. (n)**	***Escherichia coli*** **(n)**	***Staphylococcus aureus*** **(n)**	***Staphylococcus schleiferi coagulans*** **(n)**	***Pasteurella multocida*** **(n)**	***Proteus mirabilis*** **(n)**	***Pseudomonas aeruginosa*** **(n)**
Percentages shown are of all isolates (n = 194) included in the study.		(90) 46%	(47) 24%	(21) 11%	(15) 8%	(7) 4%	(6) 3%	(4) 2%	(4) 2%
**Submission categories** ^**a**^									
A	(23) 12%	11	6	1	1	2	0	1	1
B	(53) 27%	26	10	6	5	2	3	0	1
C	(64) 33%	33	16	7	4	1	2	1	0
D	(22) 11%	10	7	2	0	1	1	0	1
E	(32) 17%	10	8	5	5	1	0	2	1
**Hospitalization time categories** ^**b**^									
≤1 day	(148) 76%	68	37	16	13	6	4	2	2
≥1 day	(43) 22%	20	9	5	2	1	2	2	2
Uknown	(3) 2%	2	1	0	0	0	0	0	0
**Location** ^**c**^									
Superficial skin infection	(78) 40%	42	19	3	4	3	3	2	2
Deeper	(116) 60%	48	28	18	11	4	3	2	2
**Surgical procedure categories** ^**d**^									
Clean	(139) 72%	66	30	11	14	6	6	4	2
Clean- contaminated	(19) 10%	9	6	3	1	0	0	0	0
Dirty	(36) 18%	15	11	7	0	1	0	0	2
**Antimicrobial treatment categories** ^**e**^									
No treatment	(132) 68%	64	35	8	9	4	6	4	2
Treatment	(62) 32%	26	12	13	6	3	0	0	2

The pathogens presented by the defined categories for sample origin (submission category), hospitalization time (hospitalization time category), depth of infection (location category), surgical procedures (surgical procedure category) and whether antimicrobial treatment had been administered or not prior to sampling (antimicrobial treatment category).

^a^The four enrolled referral hospitals are labeled A, B, C and D. The three animal clinics are labeled E.

^b^Isolates from dogs with SSI that spent one day or less in the hospital or clinic are labeled hospitalization group one. The remaining isolates are labeled hospitalization group two.

^c^Surgical site infections described by the attending veterinarian as superficial and only involving skin are labeled superficial skin infections, and all infections described as either deep or deep subcutaneous are labeled deeper, as are infections including soft- or skeletal tissue and abscesses.

^d^The surgical procedures are categorized as clean, clean-contaminated, contaminated or dirty according to the classification scheme developed by The Centers for Disease Control and Prevention National Nosocomial Infections Surveillance (7).

^e^The two categories of antimicrobial treatment are labeled treatment or no treatment, according to whether antimicrobial treatment had been administered or not prior to sampling.

The same univariable tests described above were used for assessment of possible associations between the explanatory variables and whether more than one pathogen was present or not in the cultures (mixed cultures). All statistical analyses were performed using Stata Software (StataCorp., 2013; Stata Statistical Software: Release 13.1; College Station, TX, USA: StataCorpLP).

## Results

### Distribution of pathogens

In total, 157 of 203 cases of SSIs submitted during the study period met the set inclusion criteria. The remaining 46 samples yielded either no growth or only insignificant non-specific growth. In 37 (23%) of the positive cultures two pathogens were detected, leaving in total 194 pathogenic isolates from the 157 dogs included in the study. Eight different bacterial species were identified in the 194 samples. *Staphylococcus pseudintermedius* was the most prevalent finding (46%, n = 90), followed by beta haemolytic *Streptococcus* spp. (24%, n = 47), *E. coli* (11%, n = 21) and *S. aureus* (8%, n = 15). The remaining pathogenic isolates were *Staphylococcus schleiferi coagulans* (4%, n = 7), *Pasteurella multocida* (3%, n = 6), *P. mirabilis* (2%, n = 4) and *P. aeruginosa* (2%, n = 4).

An additional pathogen was identified in 66% (n = 31) of the 47 cultures positive for beta haemolytic *Streptococcus* spp., and in 30% (n = 27) of the 90 cultures positive for *S. pseudintermedius*. The two species were found in 73% and 62%, respectively, of the 37 cultures where two pathogens were detected. Two of the four *P. mirabilis* isolates, three of seven *S. schleiferi* (42%), approximately a third of the *S. aureus* and *P. multocida* isolates (30%, n = 5 and 33% n = 2, respectively), and 19% (n = 4) of the *E. coli* isolates were found in mixed cultures. It was significantly more common to find beta haemolytic *Streptococcus* spp. than S*. pseudintermedius*, *S. aureus, E. coli* and *Pseudomonas aeruginosa* in mixed cultures (P < 0.05).

Each of the four animal hospitals (A, B, C and D) submitted 11-32% of the included cases and 11-33% of the pathogenic isolates. The three animal clinics (submission category E) contributed with 29 cases and 32 (17%) isolates (Table 2). The pathogens are presented by submission category, duration of hospitalization, wound location and the three categories of surgical procedures in Table 2. Six groups of surgical procedures contributed to 92% (n = 144) of the 157 cases; abdominal surgery (n = 42; 27%), orthopaedic surgery (n = 31; 20%), mastectomy (n = 31; 20%), extirpation of subcutaneous masses (n = 23; 15%), male dog neutering (n = 10; 6%) and traumatic injuries including bite-wounds (n = 7; 4%).

### Risk factor analysis

There were no associations between distribution of the isolated pathogens and submission category, duration of hospitalization or classification of the surgical procedure. Furthermore, there were no associations between any of the explanatory factors and whether the culture was mixed with two pathogens or not. One significant association between isolated pathogen and depth of infection category was found. *Escherichia coli* was significantly more often found in deep wound infections than in superficial skin infections compared to any other bacterial species (OR = 4.7, *P* = 0.02).

### Antimicrobial treatment and susceptibility patterns

The majority of the isolates (68%; n = 132) were from cases that had not received antimicrobial treatment prior to sampling (Table 2). All of the *P. multocida* and *P. mirabilis* isolates, approximately half of the *S. schleiferi* (n = 4) and *P. aeruginosa isolates* (n = 2) isolates and two-thirds of *S. aureus* isolates belonged to the untreated group. Further statistical assessment of influence of antimicrobial treatment on pathogenic growth was not performed for these bacterial species due to the low number of isolates.

Duration of treatment prior to sampling varied from perioperative treatment during less than one day (n = 14) to more than one week (n = 29) (Table 3). Sixty-eight percent (n = 42) of the 62 isolates in the treated group were susceptible to the antimicrobial used. Susceptibility to the antimicrobial used could not be determined for four isolates due to BPs for susceptibility being outside the range of concentrations tested, and the results of the susceptibility testing of one of the *E. coli* isolates was not available. The majority (60%; n = 9) of the 15 resistant isolates in the treated group were *E. coli* (Table 3).

**Table 3:** **Isolated pathogens presented by antimicrobial treatment**

	**Antimicrobial treatment (n)**		**Susceptibility to the antimicrobial used (n)**			**Duration of treatment (n)**			**Isolates from cases treated at time of sampling (n)**	**Susceptibility to the antimicrobial used (n)**		
	**No treatment**	**Treatment**	**Susceptible isolates**	**Resistant isolates**	**Not determined**	**≤1 day**	**≤1 week**	**≥1 week**		**Susceptible isolates**	**Resistant isolates**	**Not determined**
***Staphylococcus pseudintermedius***	(64)	(26)	(20)	(5)	(1)	(5)	(5)	(16)	(13)	(10)	(3)	(0)
**beta haemolytic** ***Streptococcu*** **s spp.**	(35)	(12)	(12)	(0)	(0)	(2)	(3)	(7)	(8)	(8)	(0)	(0)
***Escherichia coli***	(8)	(13)	(1)	(9)	(3)	(0)	(9)	(4)	(9)	(0)	(8)	(1)
***Staphylococcus aureus***	(9)	(6)	(6)	(0)	(0)	(6)	(0)	(0)	(0)	(0)	(0)	(0)
***Staphylococcus schleiferi***	(4)	(3)	(2)	(1)	(0)	(1)	(1)	(1)	(1)	(0)	(1)	(0)
***Pasteurella multocida***	(6)	(0)	(0)	(0)	(0)	(0)	(0)	(0)	(0)	(0)	(0)	(0)
***Proteus mirabilis***	(4)	(0)	(0)	(0)	(0)	(0)	(0)	(0)	(0)	(0)	(0)	(0)
***Pseudomonas aeruginosa***	(2)	(2)	(1)	(0)	(1)	(0)	(1)	(1)	(2)	(1)	(0)	(1)
No. of isolates, percentages in brackets	(132) 68%^a^	(62) 32%^a^	(42) 68%^b^	(15) 24%^b^	(5) 8%^b^	(14) 22%^b^	(19) 31%^b^	(29) 47%^b^	(33) 17%^a^	(19) 58%^c^	(12) 36%^c^	(2) 6%^c^

Isolated pathogens presented by antimicrobial treatment; no treatment or treatment administered, susceptibility or resistance of respective isolate to the antimicrobial used, duration of treatment and whether treatment was ongoing at time of sampling or not.

^a^Percentage of all of the 194 pathogenic isolates included in the study.

^b^Percentage of the 62 isolates from dogs that had received antimicrobial treatment prior to sampling.

^c^Percentage of the 33 isolates from dogs that received antimicrobial at time of sampling.

Seventeen percent (n = 33) of all isolates were from cases sampled during ongoing antimicrobial treatment. Of these, 36% (n = 12) were resistant to the respective antimicrobial used (Table 3). *Escherichia coli* was significantly more often found in samples collected during ongoing antimicrobial treatment compared to the other pathogens (OR = 4.7, P = 0.002).

The results from susceptibility testing of *E. coli*, *Streptococcus* spp., *P. multocida* and staphylococci are summarized in Table 1 as the percentage susceptible isolates and as MIC_50_ and MIC_90_, i.e. the MIC required to inhibit 50% and 90% of the isolates, respectively. For some combinations of antimicrobials and bacterial species the percentage of susceptible isolates could not be determined due to CLSI BPs being outside of concentration ranges of the test panels used. MIC_90_ was not calculated for the two species *P. multocida* and *S. schleiferi* due to the small number of isolates. One isolate of *E. coli* was not available for susceptibility testing.

Almost all (95%) of the 20 *E. coli* isolates were resistant to cephalothin. Although the BPs for ampicillin was outside of concentration ranges of the test panels used, the high ampicillin MIC_50_ indicates a large proportion of ampicillin resistant isolates. The majority (80%, n = 14) of the *E. coli* isolates were susceptible to the other six antimicrobials tested. One isolate was resistant to cefotaxime and tetracycline and confirmed as an ESBL producer carrying the *bla*_CTX-M-1_ gene. No isolate had an MDR phenotype.

All of the 47 beta-haemolytic *Streptococcus* spp. isolates were resistant to gentamicin and 83% were resistant to enrofloxacin (Table 1). The percentage of susceptible isolates to ampicillin, clindamycin and erythromycin could not be determined due to CLSI BPs being outside of concentration ranges of the test panels used. However, the MIC_90_ values for these antimicrobials are truncated at the lowest tested concentration, which indicates that most isolates might be susceptible. Moreover, only one isolate had clindamycin and erythromycin MICs above the respective BP for resistance. All isolates were susceptible to penicillin and trimethoprim-sulfamethoxazole. Sixteen isolates (34%) were either intermediate or resistant to tetracycline. Disregarding enrofloxacin and gentamicin, only one beta-haemolytic *Streptococcus* spp. isolate had an MDR phenotype; the isolate was resistant to tetracycline, clindamycin and erythromycin. The six *P. multocida* isolates were susceptible to most antimicrobials tested but susceptibility to clindamycin and erythromycin could not be determined as there are no BPs for these substances.

Eighty per cent of the *S. pseudintermedius* isolates were resistant to penicillin and ampicillin. Susceptibility to the other antimicrobials varied (73–96%) (Table 1). Twenty-six percent (n = 23) were MDR. Of these, 17 were resistant to penicillin, erythromycin and clindamycin in addition to other antimicrobials. Four isolates had oxacillin MIC > 0.5 mg/L indicating methicillin resistance and in three of these the *mecA* gene was identified. The three isolates were from dogs treated at three different clinics. The methicillin-resistant isolates were all resistant to enrofloxacin, erythromycin, clindamycin and trimethoprim-sulphamethoxazole in addition to beta-lactam resistance. Most (80%) of the 15 *S. aureus* isolates were resistant to penicillin and ampicillin, one of which was in addition resistant to tetracycline (Table 1). No *S. aureus* isolate was MDR. All *S. aureus* isolates had oxacillin MICs < 0.5 mg/L. Two of the seven *S. schleiferi coagulans* isolates were MDR. All seven isolates had oxacillin MICs < 0.5 mg/L (Table 1). The percentage of staphylococcus isolates susceptible to ampicillin, amoxicillin-clavulanic acid and clindamycin could not be determined due to CLSI BPs being outside of concentration ranges of the test panels used. However, as the staphylococci were resistant to penicillin and ampicillin through beta-lactamase production, amoxicillin-clavulanic acid would most likely be a relevant antimicrobial for the isolates susceptible to cephalothin (96% of the *S. pseudintermedius* isolates, 100% of the *S. aureus* and *S. schleiferi coagulans* isolates respectively).

The four *P. aeruginosa* isolates were all susceptible to gentamicin (MIC ≤2 mg/L). Breakpoints are not available for the other antimicrobials tested. However,except for enrofloxacin where all four isolates had MIC 1 mg/L, MICs were high and mostly above the range of concentrations tested (Table 1). Breakpoints for susceptibility are largely lacking also for *P. mirabilis* but MICs for trimethoprim-sulphamethoxazole, gentamicin and enrofloxacin were low in all four isolates, ≤ 0.5/9.5, ≤ 2 and ≤ 0.12-0.25 mg/L, respectively. Moreover, three of the isolates had ampicillin MICs ≤1 mg/L. For the other antimicrobials tested MICs for were high and mostly above the range of concentrations tested (Table 1).

## Discussion

The eight bacterial species isolated in this study are known opportunistic pathogens that can be found both in healthy dogs as part of the normal skin microbiota and in various canine infections, including SSIs [1,3,30-32]. Fifty-eight percent of the 194 isolates were coagulase- positive staphylococci, all recognized as important pathogens in canine dermatitis and SSIs. The most prevalent finding, *S. pseudintermedius,* is considered to be the primary cutaneous pathogen of dogs and a well-known cause of postoperative wound infections in dogs [30,31,33,34].

In humans, the patients’ endogenous flora is the major source of bacteria infecting surgical wounds [1-3]. The endogenous skin flora is also recognized as an important cause of SSI in dogs, as aseptic preparation of the skin cannot completely eliminate skin-associated bacteria, especially not bacteria residing in the deeper parts of the skin such as the hair follicles and sebaceous glands. Both superficial and deeper infection can be the result, as the bacteria can enter deeper tissues during the initial incision [1-3,7]. All the bacteria cultured in the study, including those less commonly associated with skin disease and skin carriage than staphylococci, might be endogenous flora recently transferred to the skin at the incision site through the dogs grooming behaviour, or through contamination of affected tissues during surgery [1,7,30,35].

The different bacterial species causing the SSIs might also be exogenous microorganisms transferred from the environment. The frequency of exogenous versus endogenous flora causing SSIs on dogs is not well described [1,2,7,15]. The risk of nosocomial infections with canine opportunistic pathogens transmitted through animal clinics and hospitals has however been highlighted lately, particularly with the increase in antimicrobial resistant canine infections and the emergence of multidrug resistant clones MRSP, as well as with MRSA infections and ESC-resistant Enterobacteriaceae [36]. Exogenous sources of surgical contamination include the surgical equipment, the physical environment and bacteria on hands and clothes of personnel [1,35,37,38]. Outbreaks of MRSP infections have also been attributed to carriage of the organism in the nasal passages of clinicians and hospital workers [39]. Nosocomial pathogens have been shown to persist in the hospital environment in various locations and bacterial colonization of human hospital patients by endemic hospital organisms has been shown to occur in patients within a few days of hospitalization [35,39,40]. Due to the possibility of prolonged contact with the microbiota in the healthcare environment influencing pathogenic growth, duration of hospitalization as well as submission category was included in the present study, but no influence on distribution of the eight bacterial species was shown.

When evaluating SSIs an objective definition is desirable, also for facilitating comparison of veterinary studies, where various definitions have been used for postoperative SSI in different studies [2]. A standard classification of the wounds such as superficial incisional, deep incisional or organ/space was however deemed not appropriate for the present study as, with the exception of the infections classified as superficial, the information attained from the attending veterinarians was decided not to be rigorous enough. Infections including soft- or skeletal tissue or infections in the form of an abscess could have been classified as deep incisional, but as only seven isolates originated from this category with four bacterial species represented, the use of only the two terms superficial skin infection and deeper infection was deemed more stringent.

The National Nosocomial Infections Surveillance criteria [7] were developed for surveillance of SSIs in humans, and although a clear correlation between the four categories of surgical procedures and SSI rates has been reported in human medicine, it is not possible to determine either the risk of SSI to develop or the source of the bacteria causing the SSIs solely based on which category the surgery belongs to [2,3]. However, the risk of an SSI being caused by an infecting agent already present prior to surgery is increased in the dirty category, to which 18% of the 194 isolates belonged. Furthermore, the risk of the SSI being caused by endogenous flora from the gastrointestinal, genitourinary or respiratory tracts can be expected to be decreased in the clean category, to which 72% of the isolates belonged as procedures where these body sites are opened are not included in the clean category. No such correlations were however found in the present study. The only significant association between distribution of the isolated pathogen and the investigated explanatory factors was between growth of *E. coli* and depth of infection as well as with antimicrobial treatment during time of sampling. The reasons for these findings are unknown. The multifactorial nature of SSI, including critical factors such as adherence to aseptic principles and proper aseptic preparation and postoperative care in veterinary care facilities as well as in the home environment, complicates studies like this and further studies on associations between individual bacterial species causing SSIs and factors that can influence this are warranted.

The selection of antimicrobials for treatment of SSIs should be based on bacterial culture and susceptibility testing, particularly as SSIs and other nosocomial infections are increasingly complicated by the emergence of MDR pathogens in veterinary medicine. Susceptibility testing is a corner stone of efficient and prudent use of antimicrobials, an important step in reducing the emergence of antimicrobial resistance. Empirical treatment is however often necessary before culture and sensitivity results are available, and further studies on susceptibility patterns in nosocomial infections on dogs such as SSIs are warranted, to provide a basis for both empirical treatment and surveillance of local and general increases or decreases in the overall burden of MDR pathogens [1,2,8]. The isolates included in the present study are from both referral hospitals and smaller clinics. The enrollment of several hospitals and clinics reduces the risk of overrepresentation of the resident house flora, including susceptibility patterns, of any individual care facility. It has previously been stated that, as referral hospitals are more likely to have a higher caseload of complicated cases in need of prolonged antimicrobial treatment, less susceptible isolates should be expected in samples originating from referral hospitals compared to smaller clinics [3,41-44]. Associations between antimicrobial treatment and isolating resistant bacteria from SSIs are beyond the scope of this study, and statistical assessments of trends was not possible due to too few resistant isolates originating from cases treated for longer than one day.

Overall the results of the susceptibility testing were favourable in comparison to other studies [4,37,45-49]. The isolated pathogens were mostly without acquired antimicrobial resistance and MDR was uncommon, with the exception of staphylococci. A majority of the staphylococci isolates were resistant to penicillin and ampicillin and approximately one-fourth (26%) of all *S. pseudintermedius* isolates were MDR. Methicillin-resistant staphylococci such as MRSP and MRSA are globally reported as important causes of clinical infections, including SSIs, in dogs [3,4]. Despite a documented clonal spread of MRSP in Europe and the increasing clinical importance of the pathogen, MRSP was a rare finding in the nosocomial infections investigated in the present study [6,50].

The emergence of ESC- resistant Enterobacteriaceae in dogs not only complicates therapy in dogs, it is also a public health concern when the pathogens are zoonotic or the location of the resistance genes enables transfer between bacteria of animal and human origin [36,51]. In the present study one of 20 *E. coli* isolates tested carried transferable genes conferring ESC resistance by ESBL production. The small number of isolates tested precludes conclusions on prevalence but the finding supports vigilance towards ESC-resistance in the investigated dog population.

## Conclusions

*Staphylococcus pseudintermedius* was the most frequent pathogen identified in the study (46% of all isolates), and approximately two-thirds of all isolates were staphylococci. *Escherichia coli* isolates were significantly more often found in deep wound infections than in superficial infections. No other associations were found between the distribution of pathogens and the included explanatory factors.

Overall the possibilities of finding first line antimicrobials to treat SSIs were favorable. Except for staphylococci the isolated pathogens were mostly without acquired resistance and MDR was uncommon. As resistance in staphyloccci to penicillin and ampicillin was through beta-lactamase production, the isolates were accordingly mostly susceptible to first generation cephalosporins and most likely also to a combination of amoxicillin and clavulanic acid although this was not tested.

There were only three cases of MRSP infections (one percent of all isolates), one case of ESBL producing *E. coli*, and no MRSA infections.
